# Acne Vulgaris and Its Association with Dietary Habits at a Tertiary Care Hospital in Karachi, Pakistan

**DOI:** 10.4314/ejhs.v33i2.20

**Published:** 2023-03

**Authors:** Tafazzul Hyder Zaidi, Mubashir Zafar, Bahram Khan Khoso, Rabia Ghafoor, Saba Asghar, Saima Parveen, Usaid Ahmed

**Affiliations:** 1 Community Medicine Department, Jinnah Sindh Medical University, Pakistan; 2 Family and Community Medicine Department, College of Medicine, University of Hail, Hail, Kingdom of Saudi Arabia; 3 Department of Dermatology Jinnah Postgraduate Medical Centre/ Jinnah Sindh Medical University

**Keywords:** Acne, diet, patients, GI symptoms, oily food, self-esteem, practices, Pakistan

## Abstract

**Background:**

Acne is a common skin disorder among younger age group. Dietary pattern are the key determinants among acne patients. The objective of this study is to determine the association of acne vulgaris with dietary habits among patients at tertiary care hospital in Karachi, Pakistan.

**Methods:**

it is a cross sectional study and participants were selected through simple random sampling from outpatient department of tertiary care hospital in Karachi, Pakistan. Patients presenting with acne irrespective of severity and treatment were included in the study. Bivariate analyses conducted at 95% CI and p=<0.05 considered statistically significant.

**Results:**

Females gender (62%) and family history (61%) was significantly associated with acne. Face region was most (92%) affected by acne. GI upset most common co-morbid illness with Acne (p=0.006). After adjustment of covariate, those consume oily food [Daily oily food; Twice a weekly oily food)]; sugar rich food [Daily consumed sugar; Twice a weekly consumed] and less glass of water [8–10 glass per day; 6–8 glass per day; less than 6 glass per] were significantly associated with acne.

**Conclusion:**

Study found that un-healthy diet is significantly associated with acne. Common dietary predictors for acne were frequent use of oily food, sugary food, and less amount of drink water.

## Introduction

Acne is a common inflammatory skin disorder due to of multi-factorial origin. Global prevalence of acne approximates 9.4%, it is the eighth most prevalent disease worldwide (1). In Global Burden of Disease 2013, productivity loss and treatment costs for acne in United States crossed 1.2 billion dollars [2]. Skin related disorders were responsible for 41.6 million disability Adjusted Life Years (DALY) and 39 million years lived with disability (YLD) (2).

Acne is common among adolescents and the adults' age group. Acne is most prevalent at 16 to 18 years of age. Prevalence of acne is 75% to 98% in young age group [2]. This skin disorder is common among both gender and all ethnicity (3).

It is predicted that diet is the important determinants of acne after hormones and genetics (4). The role of “Western style diet” (a diet rich in saturated fat, large quantity of processed carbohydrate including a high calorie amount) has been found major contribution in inflammatory skin diseases such as acne, atopic dermatitis, and psoriasis (5). Research studies over the past decade have strongly reinforced the theory of gut-brain-skin axis. Altered gut flora can give rise to various inflammatory and autoimmune skin disorders(6). Gastrointestinal (GI) symptoms in patients of acne also evaluated in some studies revealing convincing results. According to different studies, mechanisms underlying the pathogenesis of acne which involved gastrointestinal dysfunction and this lead to increases sebum production(7).

Diets having high glycemic indices and dairy food like milk especially skimmed milk have shown prominent role in the severity of acne. They are enhancing the signaling of insulin like growth factor-1 (IGF-1) (8). Daily water intake has also been known to concern with skin's physiology and dermatological abnormalities. The role of dietary fiber intake has also been reported in reducing acne but no study confirmed that fiber associated with acne(8). Acne not only presents with physical morbidity but also cause psychological harm to the patient(9). Different studies carried among acne patients found that mental health disorder among acne patients(7–9). However, data reporting dietary practices among acne patients of Pakistani descent is limited. In these study sociodemographic risk factors, dietary habits, and general practices of acne patients have been studied.

## Methods

The study design was cross-sectional. Patients attending the outpatient department (OPD) of Dermatology at tertiary care hospital were included in the study. All patients between 18–45 years of age presenting with acne regardless of treatment status and severity were included as study participants. Whereas those not giving consent and presenting with skin lesions other than acne kept in the exclusion criteria. Institutional Review Board (IRB) of JPMC approved the study against Reference No. F.2-81-IRB/2019-GENL/19830/JPMC.

Sample size was calculated with Open EPI calculator for sample size. The parameter for size calculation is the knowledge level regarding acne was 65% from the previous study(10), 95% confidence interval (CI) and ±5% margin of error were used. The required sample size was computed as 378. Patients were selected through simple random sampling technique.

Pre-tested structured questionnaire was used for data collection. The outcome for pretest was that responses from participants were consistent. The questionnaire included socio-demographic information like age, gender, and marital status. Questions related to the onset of acne, family history, areas affected by acne, knowledge about causation of acne, dietary practices, general attitude, and physical activity were asked.

Each patient was interviewed by the investigators who were adequately trained to minimize the investigator bias. The answers for each question were recorded on a separate form for each patient. Data entry and analyses were carried out using SPSS software (IBM Corp.), version 26.Data from questionnaire were encode into database by data encoder. Questionnaire forms and database were checked for completeness daily. Data were entered twice and then cleaned for any missing variables. All the data were supervised by principal investigator on regular basis. Descriptive analysis was done for base line characterizes of patients, frequencies and percentages were calculated. Association between the Acne and associated risk factors were determined by logistic regression analysis. *p* value <0.05 was considered to be statistically significant.

**Ethical approval:** Institutional Review Board (IRB) of JPMC approved the study against Reference No. F.2-81-IRB/2019-GENL/19830/JPMC.

## Results

Mean age of acne patients was 21 years. Around two-third of patients were females n=234 (62%). About 32% males and 39% females were married. Most (73%) of the study participants were 18–25 years. Nearly 60% of patients had family history. More than one third (38%) of participants had ever smoker. More than, halve (55%) of patients had moderate acne and more than one third (31%) had severe acne. Common body part affected by acne is face (92%) and GI disorders (heartburn, constipation, acid reflux etc.) were the most frequently reported conditions between both genders (Table 1).

**Table 1:** Baseline characteristics of study participants

Characteristics	Frequency (n)	Proportion (%)
Age (mean ± SD)(years)	**21 ± 0.58**	
18–25	277	73.28
26–45	101	26.72
Gender		
Male	144	38.1
Female	234	61.9
Marital Status		
Single	241	63.8
Married	137	36.2
Family History of Acne		
Yes	227	60.1
No	151	39.9
Smoker		
Ever	144	38.1
Never	234	61.9
Acne Severity		
Mild	104	27.52
Moderate	155	41.00
Sever	119	31.48
Co-morbidity with Acne		
Gastro-intestinal disorder	150	39.68
Other minor disorder*	73	19.31
No disorder	155	41.01
Acne affected body part		
Face	350	92.59
Others body Parts**	28	7.41

* Diabetes, blood pressure, kidney disease

** Chest, limbs, back, shoulder, neck

After adjustment of covariate, those consume oily food[Daily oily food (OR 2.09, 95% CI 1.52–5.67); Twice a weekly oily food (OR 1.23, 95% CI 1.08–4.23)]; sugar rich food[Daily consumed sugar ( OR 3.79, 95% CI 2.29–7.58); Twice a weekly consumed sugar (OR 1.73, 95% CI 1.44–5.81)] and less glass of water[8–10 glass per day (OR 1.91,95%CI 2.01–8.98); 6–8 glass per day (OR 3.93,95% CI 1.74–4.37); less than 6 glass per day ( OR 5.88,95% CI 2.94–6.25)] were significantly associated with acne. [Table 2]

**Table 2:** Association of severity of Acne with dietary habits

Dietary Habits	Un-Adjusted Odd Ratio (UOR) and Confidence Interval (CI)	p-value	Adjusted Odd Ratio (AOR) and Confidence Interval (CI)	p-value
**Consume oily foods**				
Never	1		**1**	
Daily	**1.98(1.01–2.78)**	0.003	2.09(1.52–5.67)	0.002
Twice weekly	**1.06(0.97–3.56)**	0.008	1.23(1.08–4.23)	0.004
**Use dairy items regularly**				
No	**1**		1	
Yes	**0.87(0.22–2.34)**	**0.057**	0.95(0.12–3.45)	**0.087**
**Consume sugar rich foods**				
Never	**1**		**1**	
Daily	**3.21(2.01–6.98)**	0.003	**3.79(2.29–7.58)**	**0.001**
Twice weekly	**1.43(0.94–3.87)**	0.006	**1.73(1.44–5.81)**	**0.004**
**Consume fiber-rich diet**				
Never	**1**		**1**	
Daily	**0.31(0.01–2.98)**	0.092	**0.21(0.02–5.38)**	**0.034**
Twice weekly	**0.43(0.22–4.37)**	0.010	**0.13(0.04–1.67)**	**0.012**
**Glasses of water intake per** **day on average**				
>10	**1**		**1**	
8–10	**1.21(1.01–4.98)**	0.004	**1.91(2.01–8.98)**	**0.001**
6–8	**3.43(1.24–3.57)**	0.003	**3.93(1.74–4.37)**	**0.002**
<6	**5.43(2.54–3.77)**	0.011	**5.88(2.94–6.25)**	**0.001**

**Figure 1:**
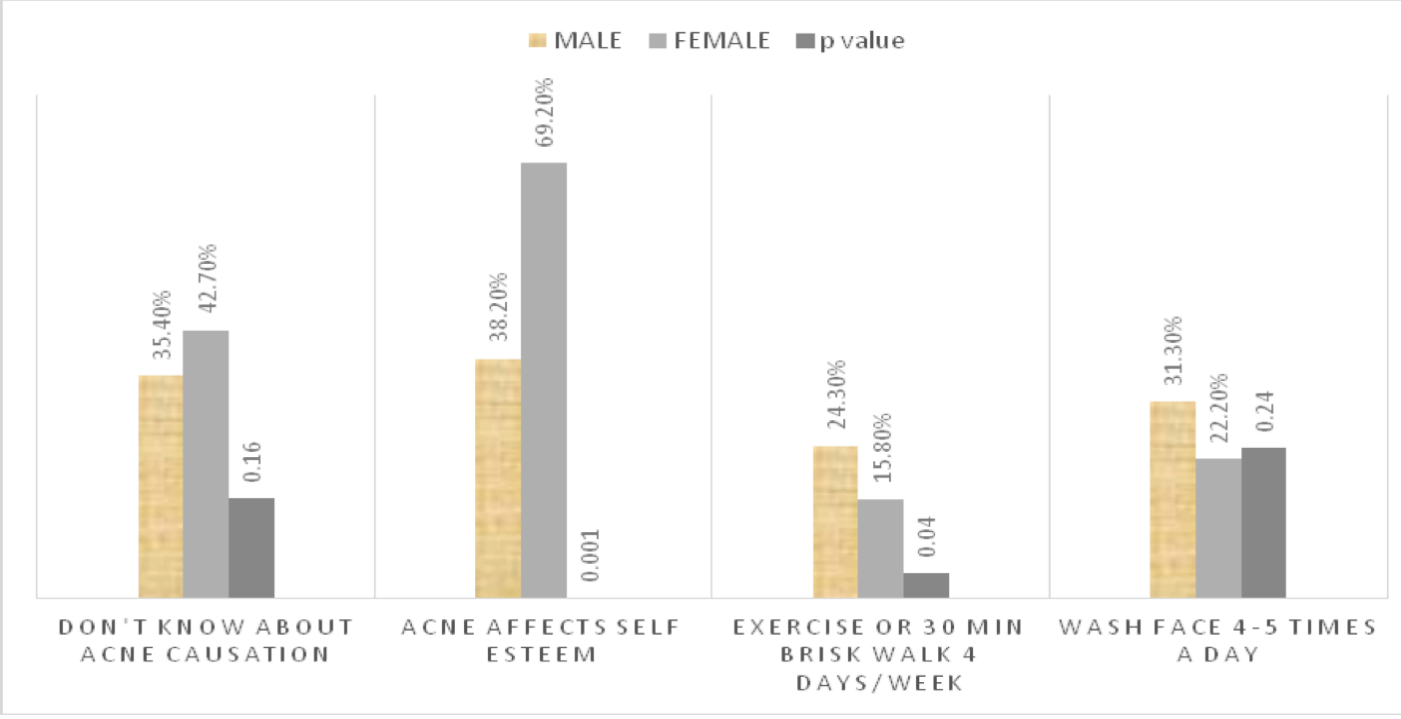
Knowledge and Attitudes among Acne Patients with respect to gender

Our patients possessed deficient knowledge about causation of acne. No significant difference found among knowledge of patients with age group (p=0.89). Upon enquiring about the effect of acne on self-esteem, females were significantly more disturbed as compared to males, 162 (69.2%) versus 55(38.2%) respectively (p=<0.001). There was insignificant difference between face hygiene practices followed by patients of different age groups (p=0.23) or opposite genders (p=0.24). Majority of male (75.7%; n=109) and female (84.2%; n=197) patients were not performing regular exercise (p=0.04), and hence, followed a sedentary lifestyle (Figure 1).

## Discussion

In this study majority of our participants were female. Similar proportions have been reported in related studies (11–12). In this study young adults were the most affected age group. Acne among same age distribution has been shown in previous literature (13–14). Acne related to age at onset of puberty were high in this study. Puberty has been strongly associated with onset of acne since sebum production is greatly enhanced along with hormonal changes(15). This creates a pro-acne environment and hence the higher prevalence rate rates among this age group. Majority of females affected by acne in our study had a positive family history for parents or siblings. A similar observation was made in a related study(16).

Most of our patients suffered from GI disorders with acne. A study was conducted which recruit 13000 adolescents, these patients have acne and co-morbid condition of gastrointestinal symptoms and common symptoms are constipation, bloating, gastric reflux, belching(17). A large U.S population-based study also showed that severity of acne is strongly associated with GI disorders like acid reflux, abdominal pain, loose stools, or chronic constipation (17). This predicts a contributory role of an ‘acne-pro’ diet by altering the natural balance of intestinal flora favoring conditions of systemic inflammation.

Cigarette smoking and areca nut (chalia) were also consumed by participants of this study. In addition to their role as potential carcinogen for lung and oral cancer, interesting correlation in aggravating acne lesions have also been reported in literature. A study conducted among 226 females found smoking as a statistically significant factor for post-adolescent acne(18).

Most of our study participants were consuming high fat diet, dairy products, and sugar rich foods. Similar findings also found in a study among acne patients from Kabul, Afghanistan (19).

In a Norwegian study conducted among adolescents showed that high intake of dairy products is significantly associated with moderate to severe acne (20). Other factors such as diet rich in free fatty acids and sugar which contributed to aggravation of acne (16–21). Fiber poor diet was consumed by the participants of our study. However, fiber rich diet has been linked with positive effects in reduction of acne (22). Daily water intake of most participants of our study was less than 5 glasses per day. Whereas an experimental study showed that 3200 ml/day intake of dietary water is related with good impact on skin physiology (23).

Our patients exhibited poor knowledge about causation of acne. Same was noted in previous studies (23–24). The reason for low level of knowledge regarding acne among acne patients was due to low level of literacy. Lower self-esteem was significantly associated with acne among young patients and female participants of this study. Several related studies demonstrated similar findings among their respondents (9,25). Hygiene practices like face washing in our study participants was not up to the mark. Despite large contradictions, a systemic review has shown some evidence for face washing in reducing acne (26). Our patients followed a sedentary lifestyle. Physical activity and exercise have been promoted for various physical, psychological, and therapeutic benefits. Likewise high BMI and low physical activity have been strongly linked with severity of acne (27–28).

This study has certain unavoidable limitations due to its study design. Our study is a single-centered study hence findings cannot be generalized to whole population. However, participants of our study exhibited risk factors potentiating development of acne. Experimental studies are required for demonstrating definite causative role of these factors in etio-pathogenesis of acne.

Dietary risk factors among acne patients were assessed. Majority were consuming a high fat, sugar rich and fiber poor diet. Dairy products were also consumed frequently. Most patients had a sedentary lifestyle. All these dietary components along with family history and physical inactivity which were found in our study population are well documented risk factors exhibiting either causative or aggravating role in acne. Patients should be educated about aggravating factors related to their condition.
